# Understanding the brain by controlling neural activity

**DOI:** 10.1098/rstb.2014.0201

**Published:** 2015-09-19

**Authors:** Kristine Krug, C. Daniel Salzman, Scott Waddell

**Affiliations:** 1 Deparment of Physiology, Anatomy and Genetics, University of Oxford, Oxford, UK; 2 Centre for Neural Circuits and Behaviour, University of Oxford, Oxford, UK; 3 Departments of Neuroscience and Psychiatry, Columbia University, New York, NY, USA; 4 New York State Psychiatric Institute, New York, NY 10032, USA

**Keywords:** brain stimulation, behaviour, perception, optogenetics, neurophysiology, neural circuits

## Abstract

Causal methods to interrogate brain function have been employed since the advent of modern neuroscience in the nineteenth century. Initially, randomly placed electrodes and stimulation of parts of the living brain were used to localize specific functions to these areas. Recent technical developments have rejuvenated this approach by providing more precise tools to dissect the neural circuits underlying behaviour, perception and cognition. Carefully controlled behavioural experiments have been combined with electrical devices, targeted genetically encoded tools and neurochemical approaches to manipulate information processing in the brain. The ability to control brain activity in these ways not only deepens our understanding of brain function but also provides new avenues for clinical intervention, particularly in conditions where brain processing has gone awry.

## 1. From localization of brain function with electrical stimulation to manipulating behavioural, perceptual and cognitive processes

Electrical stimulation of parts of the living brain and lesion studies—such as those of Paul Broca—were among the very first techniques to demonstrate specialization of neural function. In 1870, long before neural recording techniques became sophisticated enough to correlate cortical activity with inputs and outputs, Fritsch & Hitzig [1] concluded in their landmark stimulation study in dogs:

… sondern das vielmehr sicher einzelne seelische Functionen, wahrscheinlich alle, zu ihrem Eintritt in die Materie oder zur Entstehung aus derselben auf circumscripte Centra der Grosshirnrinde angewiesen sind. [1, p. 332]

… rather that certainly individual mental functions, probably all, are referred to circumscript centers of the cerebral cortex for their entrance into matter or for the coming about of matter. [2, p. 130]

This work led the way to detailed studies of the primate motor cortex by Sherrington, Penfield and others showing how the brain controls motor behaviour through a number of ordered maps of the body [3,4]. In this issue, Sirigu and Desmurget review how brain intervention studies have shaped our understanding of cortical motor control and beyond [5]. While many of the first electrical stimulation studies focused on motor behaviour in anaesthetized and sedated animals, it became clear that stimulation in other parts of the brain would be able to alter perceptual or cognitive processes in a very similar way. As Sherrington stated artfully in a 1922 lecture:

To pass from a nerve impulse to a psychical event, a sense-impression, percept, or emotion is, as it were, to step from one world to another and incommensurable one. We might expect, then, that at the places of transition from its non-mental to its mental regions the brain would exhibit some striking change of structure. But it is not so; in the mental parts of the brain there is nothing but the same old structural elements, set end to end, suggesting the one function of the transmission and collision of nerve impulses. [6, p. 350]

Since the early experiments in the motor system, landmark stimulation experiments, like for instance those by Hess, have linked electrical activation of the hypothalamus to autonomic control in the awake cat [7]. Olds & Milner's [8] electrical stimulation experiments in the septum and nucleus accumbens of rodents led these animals to repeat behaviours that would trigger further brain stimulation, consistent with experience of reward and pleasure. When homologue brain regions were stimulated in humans by Delgado [9], feelings of euphoria could be generated, reported to be so strong as to be able to supplant feelings of depression or pain.

To investigate how brain activity generates perception and cognition, direct interventions are required in subjects who can report the effect of the perturbation. Many important experiments on motor and perceptual function, like those by Penfield [4], have therefore been performed in human patients, awoken during brain surgery for treatment of epilepsy or for tumour removal. Such human experiments are, of course, only possible when there is a primary clinical need. Therefore, studies in awake-trained Rhesus monkeys have become increasingly important for investigating the neural basis of perception and cognitive behaviour. Monkeys can be trained to respond to, and make decisions about, sensory inputs or even just low levels of direct electrical stimulation of the brain (e.g. [10,11].)

Combining sensory and electrical stimulation allows the dissection of neural and behavioural patterns in a much more tightly controlled way [10,12,13]. This approach has made a particular contribution to our understanding of how physiologically classified neurons contribute to the perception of specific aspects of the visual world around us (see Cicmil & Krug in this issue [14]). Closing the loop from sensory input to behaviour, causal electrical and neurochemical methods have been combined with closely controlled behavioural tasks to dissect the primate brain circuits for active vision from the retina to execution of eye movements, as described in Wurtz's review [15]. The interplay between precise but invasive intervention studies in non-human primates with new intervention methods in humans, such as transcranial magnetic stimulation (TMS), allows one to address increasingly complex questions about how neural brain signals shape our experience and judgements (see Yau *et al.* [16] for a review on multi-sensory integration). Being able to link causal intervention in a neural circuit with a predictable and repeatable change in relevant behavioural judgements is one of the central criteria to assign a set of neurons to a particular perceptual or cognitive process [17]. This has been achieved for a number of aspects of visual and somatosensory function in the primate.

## 2. Development of more sophisticated tools to manipulate brain circuits

In many ways, it remains highly surprising that such a crude intervention as direct electrical stimulation of the brain can result in a measurable effect on perception or behaviour. Perhaps, focal cortical stimulation and surface electrical stimulation produce consistent behavioural or specific perceptual changes because neurons with similar response properties can be found in close proximity to one other, like for instance in cortical columns [18,19], and therefore can be stimulated together. Thus, electrical microstimulation methods have primarily been applied in brain structures exhibiting an anatomical organization with functional clusters. The relatively recent development of more sophisticated methods for causal interference, such as nanostimulation and optogenetics, provide a more precise intervention with a greater flexibility. Nanostimulation permits activation of single brain cells in awake animals, facilitating the study of the importance of patterned electrical activity (reviewed by Doron & Brecht [20]). Genetics provides a replicable cellular precision that is otherwise impossible. Neurons can be selectively controlled based on unique gene expression, rather than just their location relative to a stimulating device. One can then use opto-, chemo- or thermogenetic tricks to produce light-, chemical- or heat-regulated channels in these spatially disparate sets of neurons to either activate or inhibit their function [21–25]. These approaches have particular strength in simpler species, like *Caenorhabditis elegans* and *Drosophila*, where taken with the reduced numerical complexity of the nervous system, one can functionally dissect entire brain circuits and determine how they interact to generate different behavioural patterns (see Fang-Yen *et al.* [22] for a detailed review of *C. elegans*). Research in the fruit fly has provided the test-bed for much of the technical development as well as providing a defined neural platform to investigate fundamental neural operations underlying memory, reward, motivation and decision-making (see Oswald *et al.* [23] for a review). Optogenetics has also been employed in rodents to permit the study of reward, anxiety and emotional responses, which has potential for understanding the psychopathology of addiction and a variety of other psychiatric disorders (discussed by Saunders *et al*. and Gore *et al*. [24,25]).

Neural electrophysiology studies in monkeys suggest that even simple sensory stimuli generate neural responses across many areas of the brain. Therefore, intervention methods must be considered within the context of this potentially widespread neural activity, and the complex temporal interactions of feed-forward and feedback signals that must arise within, and between, local brain circuits. Using genetic strategies, neurons can now be targeted that, for example, project or receive input from a specific brain area or are activated in a specific context. In contrast to electrical stimulation approaches, these neurons can be selectively activated and inactivated even when they are locally intermingled with other neurons (see Gore *et al.* [25] for a critical review of these approaches). But it remains to be seen whether combinations of functionally, genetically, anatomically and perhaps morphologically targeted intervention methods will succeed in identifying and controlling the circuitry underlying complex cognitive behaviours. Particularly in the primate brain, such experimental approaches are still relatively hampered by the limited available ways to gain cellular specificity, by the complex response patterns often observed in single neurons and, especially, by widespread activity patterns that present significant challenges for generating complex patterns of activation across a disparate group of neurons.

## 3. Opportunities and implications for clinical practice

Being able to alter the way that humans and animals experience and respond to their environment brings with it tremendous opportunities and responsibilities. While we are far from ‘electronic mind control′ [9], deep brain stimulation already represents a considerable advance in the treatment of Parkinsons patients, and cochlear implants have been used successfully to treat some forms of deafness. Researchers and clinicians are developing neural prostheses that can interact directly with the brain to either transmit sensory information gathered by an electronic device or communicate with de-afferented or even artificial limbs. A tight interaction between research and the clinic is essential for these translational developments. For instance, developing effective retinal prostheses for blind people requires an understanding of how different electrical stimulation patterns in the retina might be read out by the brain and what kind of visual percept is generated by different patterns of stimulation (see Fine & Boynton [26]). Similarly, effective control of robotic limbs requires somatosensory feedback and an understanding of how patients can learn to sense artificial limbs (discussed by Bensmaia [27]). The advent of viable brain–computer devices puts the restoration or partial replacement of lost functions firmly on the agenda. Such developments will in turn generate insight into how different electrical patterns introduced into the nervous system specifically shape perception, behaviour and cognition.

Of course, the long history of neurochemical treatments for psychological disorders shows that brain activity can be altered in other ways. The paper by Warren *et al.* [28] discusses the effect of neurochemical treatments on emotional disorders such as depression and anxiety, both in terms of the effects on behaviour and the brain. These interventions are intrinsically more spatially distributed than electrical stimulation and many current optogenetic interventions, and one challenge lies in achieving the necessary spatial and temporal control, for example, with DREADD technology (Designer Receptors Exclusively Activated by Designer Drugs) [29]. Neurochemical treatments also work as cognitive enhancers, for example, to improve cognitive function in Alzheimer's patients, or are used to boost performance in healthy individuals. Sahakian *et al.* [30] review this topic, and they present research showing that motivation and cognition can also be affected by behavioural interventions.

If manipulating the brain with the described wide variety of neural devices, prostheses and neurochemical treatments can alter a person's behaviour, this should have an impact on our consideration of what we determine to be ‘self-control’ over own choices and behaviour. The paper by Roskies [31] discusses the challenges and conceptual hurdles to commonly held concepts of agency that are raised by direct brain intervention.

In closing, causal methods controlling brain activity have been instrumental in directly linking neural activity patterns in specific brain areas to distinct neural functions. The development of more sophisticated methods that permit interventions with improved specificity provides a strong impetus for major advances in basic brain research and in clinical practice. Our increasing understanding of brain function and the constantly evolving ability to alter it raise many philosophical and ethical questions that we will need to handle carefully as individuals and as a society.

## Author profiles

**Kristine Krug** studied Physiological Sciences at Oxford University. After a DPhil investigating the development of visual maps in cortex, she conducted research on the neural basis of motion and three-dimensional perception in primates during postdocs at the MPI in Tübingen and at Oxford University. In 2001, she was awarded a Dorothy Hodgkin Fellowship by the Royal Society to lead her own research group. She is an Associate Professor of Neuroscience at Oxford University and holds a Royal Society University Research Fellowship. Kristine's research group investigates perceptual decision-making in monkeys and humans—from single neurons to behaviour.

**C. Daniel Salzman** turned to neuroscience while a medical student at Stanford University. His PhD thesis provided causal evidence linking the activity of physiologically classified neurons in visual cortex with perceptual judgements. After graduating from Stanford with his MD and PhD, Daniel returned to the clinical arena, completing an internship and residency in psychiatry, both at Stanford University Hospital. He then resumed his research career at Stanford before joining the faculty at Columbia University, where he has become a Professor in the Departments of Neuroscience and Psychiatry. Daniel's research program combines electrophysiological, genetic and behavioural techniques to understand brain structures involved in psychiatric disorders. His particular focus concerns the neurobiology of emotion, the neural mechanisms by which cognitive processes regulate emotions, and the processes through which emotions influence cognitive functions.

**Scott Waddell** studied biochemistry as an undergraduate at the University of Dundee, and researched his PhD in cancer biology at the University of London. After postdoctoral study in the Department of Brain and Cognitive Sciences at Massachusetts Institute of Technology, he spent 10 years leading a research group in the Department of Neurobiology at the University of Massachusetts Medical School. In 2011 he became a Professor of Neurobiology and Wellcome Trust Senior Research Fellow in Basic Biomedical Sciences in the Centre for Neural Circuits and Behaviour at the University of Oxford. Scott's group studies neural transposition, and neural circuit properties of memory-motivated behaviour in the fruit fly.
